# Comparison of intermittent pneumatic compression device and compression stockings for workers with leg edema and pain after prolonged standing: a prospective crossover clinical trial

**DOI:** 10.1186/s12891-022-05975-6

**Published:** 2022-11-23

**Authors:** Da-Sol Kim, Yu Hui Won, Myoung-Hwan Ko

**Affiliations:** 1 Department of Physical Medicine and Rehabilitation, Jeonbuk National University Medical School, Jeonju, Republic of Korea; 2 Research Institute of Clinical Medicine, Jeonbuk National University–Biomedical Research Institute of Jeonbuk National University Hospital, Jeonju, Republic of Korea

**Keywords:** Intermittent pneumatic compression devices, Compression stocking, Edema, Pain, Standing

## Abstract

**Background:**

During prolonged standing, insufficient calf muscle pumping accompanies venous stasis and hypertension in the lower legs, resulting in valve dysfunction, venous wall problems, and sub-sequent inflammation. Compression therapy, which includes medical compression stockings (MCS) and mechanical intermittent pneumatic compression (IPC), is one of the most effective therapeutic interventions for treating chronic venous diseases. This study aimed to compare the therapeutic effect among resting, IPC and MCS alone, and IPC with MCS in long-standing workers (> 8 h daily).

**Methods:**

This crossover trial was conducted with 39 participants with complaints of leg edema and pain whose work involved standing for more than 8 h daily. Four treatment protocols were established for each visit as follows: protocol A (not wear MCS during work and rest without IPC after work), protocol B (wear MCS during work and rest without IPC after work), protocol C (not wear MCS during work and treat with IPC after work), and protocol D (wear MCS during work and treat with IPC after work). The primary outcome was the visual analogue scale (VAS) score for leg pain. The secondary outcomes were leg volume (mL), circumference (cm), extracellular fluid/total body fluid (ECF/TBF), and extracellular water/total body water (ECW/TBW) through bioelectrical impedance analysis. Outcomes were assessed before work (T0), after work (T1), and 60 min after intervention (T2).

**Results:**

All four protocols had significantly increased leg pain after work (T0-1) but improved 60 min after intervention (T1-2), particularly protocol C (decreased VAS by 1.9). When leg swelling was compared at T0 and T1, protocols A and C showed significant increases in leg volume and circumference, indicating significant work-induced edema, whereas protocols B and D showed no change or even a decrease. After interventions, leg volume and circumference significantly decreased in protocols A and C, although protocols B and C did not show significant improvement. The ECF/TBF and ECW/TBW of all protocols decreased after interventions.

**Conclusions:**

Leg pain and edema after prolonged standing (T1-T2) in adults were safely and effectively improved by both IPC alone and IPC with MCS. Although the use of MCS during the workday did not show improvement in leg pain immediately after work (T0-T1), both MCS with resting and MCS with IPC decreased leg pain at T1-T2 and prevented leg edema at T0-T1.

**Trial registration:**

This trial protocol was registered at the Clinical Research Information Service (KCT0005383, the date of first registration: 08/09/2020).

## Background

Many workers are required to stand for long periods of time, these workers include nurses, teachers, shop assistants, cooks, pharmacists, and hairdressers, or sit, such as office workers, drivers, and information technology specialists [1, 2]. In Dutch standing work guidelines, more than 1 h of continuous standing and more than 4 h of standing in total is unsafe and places the individual at a high risk of strain and health problems, and for which immediate intervention to reduce the strain should be performed [3]. Prolonged standing has reportedly been associated with negative health outcomes, including chronic venous diseases, fatigue, low back pain, carotid atherosclerosis, ischemic heart disease, orthostatic intolerance, and pregnancy issues, such as increased preterm birth or spontaneous abortion [4–13]. Leg immobility from prolonged standing causes ineffective calf muscle pumping and subsequent venous blood pooling and hypertension in the lower extremities [14]. These hemodynamic abnormalities in the veins of the lower legs might be related to valve dysfunction, increased venous wall tension and distension, and endothelial dysfunction and leukocyte infiltration, which lead to a cascade of inflammation [14].

Prolonged standing commonly contributes to a physiologic venous insufficiency in long-standing workers, who complain of swelling of the lower limbs, a heavy feeling in the legs, leg numbness, skin itching, dilated capillaries, varicose veins, and skin discoloration [2]. At the same time, the prevalence of pathologic chronic venous insufficiency (CVI) is higher in this group. Previous studies on 636 healthy healthcare workers suggested that 69% of hospital employees presented with clinical signs of CVI (C ≥ 1 of the Clinical, Etiology, Anatomy, and Pathophysiology [CEAP] classification), and 82% of them with newly detected venous reflux on ultrasound [1].

Treatment of venous disease consists of medication and conservative treatment, including compression by elastic stockings, bandage, and intermittent pneumatic pressure; sclerotherapy; endovenous ablation; cyanoacrylate; and surgical intervention [15, 16]. Rabe et al. demonstrated that all levels of MCS, which is the most common form of compression therapy, are recommended for the improvement of occupational leg venous symptoms, quality of life, and edema [17]. Mechanical IPC using pneumatic cuffs connected to a pump is used for limb compression therapy. IPC has been used for the prevention of deep vein thrombosis and the treatment of arterial disease, lymphedema, and CVI, especially venous ulcers [18–22]. Although both MCS and IPC have long histories of clinical application, there is no standard consensus on the frequency, pressure, duration, and combination protocol with other methods of compression and surgical intervention.

This study aimed to compare four protocols (not wear MCS during work and rest without IPC after work; wear MCS during work and rest without IPC after work; not wear MCS during work and treat with IPC after work; and wear MCS during work and treat with IPC after work) in the relief of venous symptoms of prolonged stationary participants. The primary outcome was pain score (visual analog scale, VAS), and the secondary outcomes were the leg volume and circumference. These results can provide an effective therapeutic option among the four different protocols for the improvement of venous symptoms in prolonged stationary participants.

## Materials and methods

### Study population

We recruited adults aged 19 years and older who worked in a prolonged stationary standing position at the Biomedical Research Institute and Translational Research and Clinical Trial Center for Medical Device, a tertiary university hospital, from December 2019 to April 2020. According to the “Health Guide for People who Work Standing” published by the Korea Occupational Safety and Health Agency, “jobs that require prolonged standing include salespersons or cashiers at wholesale and retailers such as large discount stores, launderers, hairdressers, workers in assembly lines, packaging industry, construction workers, healthcare employees, teachers” [23]. The inclusion criteria were: 1) current working history of prolonged standing for ≥ 8 h per day; 2) having self-reported venous symptoms, such as leg pain and swelling; and 3) fully understanding the purpose and procedures of the study, and voluntarily expressing willingness to participate. The exclusion criteria were: 1) younger than 19 years old (the age range of recruitment in South Korea is from 19 to over 80 years old); 2) cognitive impairment with difficulty in expressing the pain location and scoring using the analogue scale by themselves; 3) ankle-brachial index of ≤ 0.8 (the test was performed at the screening visit), with suspected underlying peripheral arterial occlusive disease; 4) diagnosis of deep vein thrombosis and other venous obstructive disorders on duplex ultrasound during the screening visit (venous insufficiency is not included in the exclusion criteria); 5) leg hypoesthesia; 6) recent leg surgery (within 6 months); and 7) other individually applied criteria at the discretion of the investigator. A total of 46 participants were eligible for study participation, of whom 40 were enrolled in the study, and six were excluded because they did not meet the inclusion criteria. Only one participant was lost to follow-up, and 39 participants completed the final follow-up. The mean age was 30.03 ± 7.6, and 51.3% of the participants were female (Table 1).

**Table 1:** Demographic characteristics of 39 healthy subjects who worked prolonged standing for more than 8 h per day and had leg pain and swelling

Parameter	All participants (*n* = 39)
Age – years	30.03 ± 7.6
Sex – male: female (%)	19:20 (48.7%:51.3%)
Occupations	Part-time lecturer 10 Teacher 9 Cashier 10 Health care worker 10
Ultrasonography results	
Deep vein reflux the number of subjects	35
Site of deep vein reflux – both: right: left	24: 7: 4
Ankle brachial index – mean ± SD	
Right	1.07 ± 0.08 (range 0.92–1.32)
Left	1.07 ± 0.07 (range 0.96–1.28)

### Study stocking and device

#### Standard medical compression stocking

The Stocking Simply Coton Fin (THUASNE, Levallois-Perret, France) is a commonly prescribed thigh-high MCS with closed toes exerting 23–32 mmHg pressure at the medial supramalleolar area.

#### Intermittent pneumatic compression device

We used an SMA-100 (WelbuTech, Seoul, Korea), an IPC device with five chambers which can change its pressure ranging from 0 to 200 mmHg. Identical pressure was applied to all chambers; the pneumatic compression shifts to the next chamber once the set pressure has been reached inside the previous chamber, as measured by a pressure sensor. There are two IPC modes of application (sequential and circular modes). In the sequential mode, the set pressure is initially pumped into the distal chamber; after the distal chamber is deflated, the pressure is pumped into the proximal chamber. On the other hand, in the circular mode, the distal chamber is pumped followed by the proximal chamber, without decompression of the distal chamber. After all chambers are inflated, the chambers are deflated at once.

### Study design

**Fig. 1:**
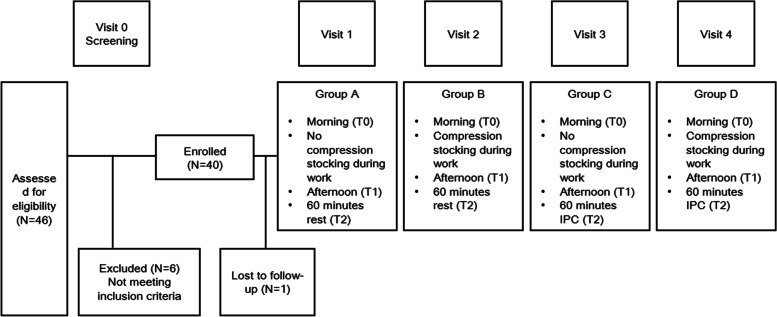
Flowchart of the procedures for the clinical trial

This clinical trial followed a crossover study design. People who worked in a prolonged stationary standing position and complained of leg pain and swelling were screened. After assessing eligibility and obtaining written informed consent, which was approved by the institutional review board of Jeonbuk National University Hospital (CUH 2019–05-064–002), participants were screened according to the inclusion and exclusion criteria (Fig. 1). Except for one subject who was lost to follow-up, 39 participants enrolled in the study following the screening completed five study visits. The occupations of the participants were part-time lecturer, teacher, cashier, and healthcare worker (mostly nurses). During the screening visit, we collected demographic data, baseline vital signs, medical histories (past histories and chronic underlying diseases), and current medications, and performed physical examination, duplex ultrasound, and ankle-brachial index test. For visits 1 to 4, measurements of leg pain (using VAS), leg volume, circumference, and bioelectrical impedance analysis were performed three times during each of the study visits (T0: morning visit before work, T1: evening visit after work, T2: 60 min after resting or interventions). All participants performed the same visit sequence Protocol ABCD, which was not blinded and randomized. For visit 1, participants worked in a standing position without wearing MCS and rested for 60 min in the supine position (Protocol A). For visit 2 (within 7 days of visit 1), MCS was used for more than 8 h during the workday and rested in the same manner as in visit 1 (Protocol B). At visit 3, they did not wear MCS and used the IPC device for a total of 60 min, with 30 min of circular and sequential modes at a pressure of 90–130 mmHg in the supine position (Protocol C). Finally, for visit 4, MCS was worn for more than 8 h during the workday and the IPC device was used similarly as in visit 3 (Protocol D). The pressure in each chamber was set by the participants within the range of 90–130 mmHg [24], such that the pressure did not cause excessive pain or discomfort. A total of nine visits were required, including the screening visit and visits 1 to 4 (within 7 days of the previous visit, comprising of 2 visits each: before and after work). Participants had to engage in work that required at least 8 h of standing. Each participant performed the standing work task assigned to their occupation at their workplace and was asked to refrain from any activity that may reduce leg pain and swelling during work, such as resting on their backs. Participants were monitored for safety issues and adverse events during work and treatment by question and answer during adverse event reporting at each visit.

### Outcome measures

#### Primary outcome

The primary outcome was leg pain evaluated via VAS, which was a continuous scale, defined as 0 for no pain and 10 for the most severe pain.

#### Secondary outcomes

The secondary outcome was leg swelling as measured by leg volume, circumference, and bioelectrical impedance analysis. We measured leg volume in each leg using the water displacement method and recorded it in milliliters [25]. Leg circumference was also measured in each leg, with the limb in a relaxed position. We measured the circumference of each foot, ankle at 2 cm above the medial malleolus, calf at 10 cm below the inferior pole of the patella, distal thigh at 10 cm above the superior pole of the patella, and proximal thigh at 20 cm above the superior pole of the patella [26]. For bioelectrical impedance analysis, we evaluated ECF, TBF, ECW, and TBW using the Inbody 3.0 system (Bio-space Co., Seoul, South Korea), which provides whole body, trunk, torso, and limb values [27, 28]. Participants stood on the Inbody 720 scale with their sole in contact with the foot electrodes as motionless as possible. Then, they grasped the handle with each palm, finger, and thumb making contact with the hand electrodes.

### Statistical analysis

All statistical analyses were conducted using the SPSS 24.0 software. For each study visit, the primary outcome (VAS) and secondary outcomes (leg volume and leg circumference) were compared across T0, T1, and T2. We compared the primary and secondary outcomes between T0 and T1 in each protocol to identify the provocation of leg pain and swelling after work in the prolonged standing position, and between T1 and T2 to verify the effect of the interventions. Normality was tested using the Shapiro–Wilk test. Because all data did not fulfill normality and sphericity, we analyzed with the nonparametric Friedman test, followed by a Wilcoxon signed-rank test, to determine whether significant differences occurred with respect to treatment interventions. Bonferroni correction was performed for the post-hoc test to ensure that the alpha was maintained at 0.05.

## Results

### Subject demographics

The baseline characteristics are presented in Table 1. Most participants were employed in the following occupations: part-time lecturers (10; 25.6%), teachers (9; 23.0%), cashiers (10; 25.6%), and healthcare workers (10; 25.6%). At the screening ultrasound examination, 35 of the 39 participants had newly detected deep vein reflux, and none had deep vein thrombosis or other obstructive arteriovenous diseases. In addition, any peripheral arterial obstruction was discovered in the ABI test.

### Pain score as the primary outcome

**Fig. 2:**
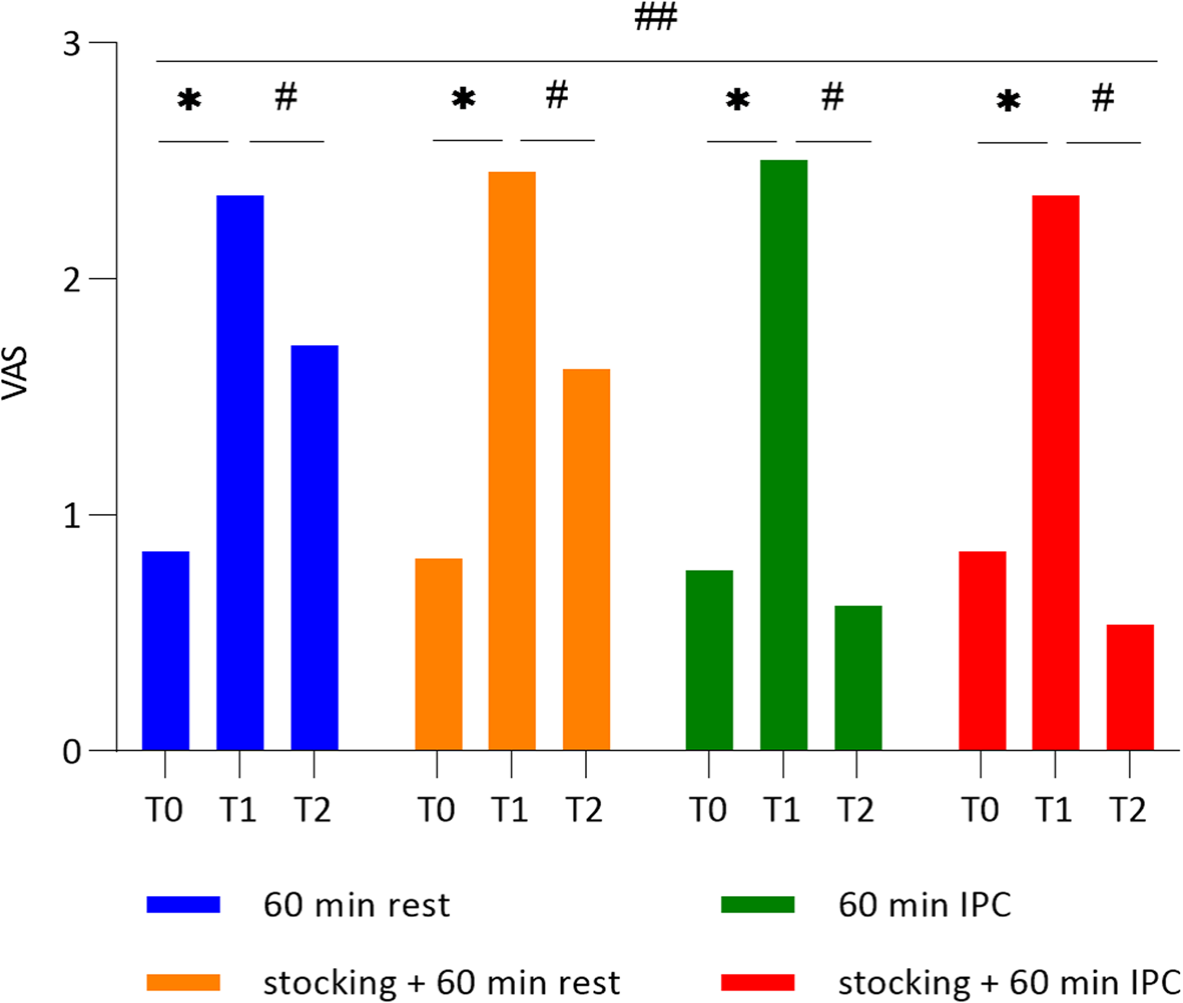
The effect of the different intervention protocols on leg pain score (Visual Analogue Scale), T0: before work, T1: after prolonged standing work, T2: 60 min after intervention, *: *p* < 0.05 between T0 and T1, #: *p* < 0.05 between T1 and T2, ##: *p* < 0.05 in the four intervention protocols

In all protocols, the pain score measured in the afternoon after work (T1) significantly increased by more than 1.5 (△pain score T1-T0 in protocol A, 1.52 ± 0.21; B, 1.64 ± 0.33; C, 1.74 ± 0.48; D, 1.51 ± 0.32) compared to that measured in the morning before work (T0), showing that leg pain was provoked at T1 by prolonged standing work (Fig. 2). After four interventions, the differences in the scores between the post-workday and post-intervention periods (T1-T2) were significant in all protocols with respect to pain reduction (△pain score T1-T2 protocol A, 0.64 ± 0.18; B, 0.84 ± 0.24; C, 1.89 ± 0.66; D, 1.82 ± 0.68). There were also significant differences among the four interventions. The post-hoc test showed differences in pain improvement between A and C, A and D, B and C, and B and D, but we did not observe any differences between A and B, and C and D (Table 2).

**Table 2:** Post hoc results of comparison between the different treatment protocols (A-B, A-C, A-D, B-C, B-D, and C-D) on leg pain as a primary outcome

Protocol	△ between the groups	*p* value	95% CI
A-B	-0.21(1.34)	0.364	-0.64 ~ 0.23
A-C	-1.26(1.55)	< 0.001	-1.76 ~ -0.75
A-D	-1.18(1.34)	< 0.001	-1.61 ~ -0.75
B-C	-1.05(1.82)	0.002	-1.64 ~ -0.46
B-D	-0.97(1.75)	0.002	-1.54 ~ -0.41
C-D	0.08(1.33)	0.831	-0.35 ~ -0.51

Data are presented as mean difference (standard deviation)

Protocol A, no medical compression stocking (MCS) + natural rest; Protocol B, MCS + natural rest; Protocol C, no MCS + intermittent pneumatic compression (IPC); Protocol D, MCS + IPC; △, the difference of leg pain score measured by visual analog scale during T1-T2 between the different treatment protocols

Wilcoxon signed rank test on difference (*p* < 0.008)

### Leg volume as a secondary outcome

**Fig. 3:**
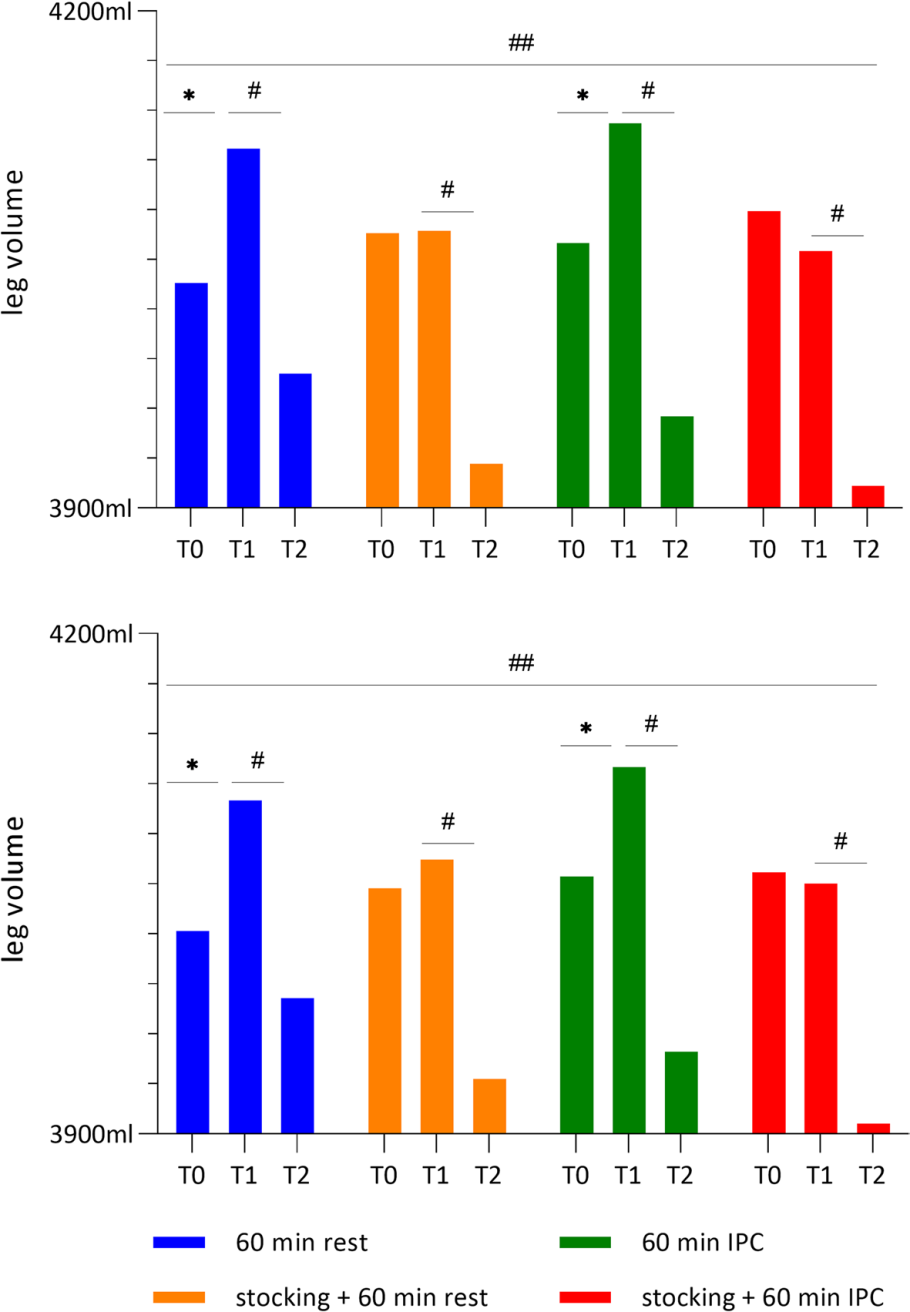
The effect of the different intervention protocols on leg volume (mL). A. Right leg, B. Left leg, T0: before work, T1: after prolonged standing work, T2: 60 min after intervention *: *p* < 0.05 between T0 and T1, #: *p* < 0.05 between T1 and T2, ##: *p* < 0.05 in the four intervention protocols

In the right leg, the leg volume of protocol A and C, who both did not wear MCS, significantly increased in the afternoon after work compared to that measured in the morning before work (△leg volume T1-T0 in protocol A, 81.1 ± 2.9; C, 72.3 ± 25.8); meanwhile, protocol B and D, who both wore MCS, showed no significant differences between T1 and T0 (△leg volume T1-T0 in protocol B, 1.6 ± 10.4; D, -24.0 ± 3.5) (Fig. 3). Despite of the prolonged standing work, the mean volume in protocol D decreased by 24.0 cc. The volume after interventions (T2) significantly decreased in all four protocols compared to those measured at T1 (△leg volume T1-T2 protocol A, 135.7 ± 6.7; B, 140.6 ± 7.3; C, 176.8 ± 14.1; D, 141.7 ± 5.9). In particular, protocol C (no MCS + IPC) showed the largest leg volume reduction. There were also significant differences across the four interventions (*p* = 0.014). In the post-hoc test, the difference in leg volume reduction was only observed between A and C, B and C, and C and D (Table 3).

**Table 3:** Post hoc results of comparison between the different treatment protocols (A-B, A-C, A-D, B-C, B-D, and C-D) on leg volume as a secondary outcome

	Protocol	△ between the groups	*p* value	95% CI
Right	A-B	-4.85(86.12)	0.675	-32.76 ~ 23.07
	A-C	-41.13(76.92)	0.002	-66.06 ~ -16.20
	A-D	-5.95(78.80)	0.884	-31.49 ~ 19.59
	B-C	-36.28(89.25)	0.012	-65.21 ~ -7.35
	B-D	-1.10(89.57)	0.643	-30.14 ~ 27.93
	C-D	35.18(73.45)	0.005	11.37 ~ 58.99
Left	A-B	-13.03(65.34)	0.270	-34.21 ~ 8.16
	A-C	-51.97(70.92)	< 0.001	-74.96 ~ -28.98
	A-D	-25.51(89.32)	0.135	-54.47 ~ 3.44
	B-C	-38.95(73.04)	0.004	-62.62 ~ -15.27
	B-D	-12.49(74.35)	0.353	-36.59 ~ 11.61
	C-D	26.46(87.74)	0.005	-1.98 ~ 54.90

Data are presented as mean difference (standard deviation) (ml)

Group A, no medical compression stocking (MCS) + natural rest; Group B, MCS + natural rest; Group C, no MCS + intermittent pneumatic compression (IPC); Group D, MCS + IPC; △, the difference of leg volume (ml) during T1-T2 between the different treatment protocols

Wilcoxon signed rank test on difference (*p* < 0.008)

Similar to the right leg, the differences in leg volume of the left leg between T0 and T1 significantly increased in protocol A and C, but those in the MCS-wearing protocol B and D did not show significance (△leg volume T1-T0 protocol A, 78.3 ± 45.0; B, 17.3 ± 21.2; C, 65.4 ± 11.1; D, -6.7 ± 10.0). In protocol D, the volume decreased by 6.7 cc in spite of prolonged standing work. The volume after the interventions (T2) in all four protocols significantly de-creased compared to that measured after the workday (T1), with the mean volume in protocol C showing the largest decrement of 170.7 cc (△leg volume T1-T2 protocol A, 118.7 ± 3.3; B, 131.8 ± 15.3; C, 170.7 ± 22.8; D, 144.3 ± 15.9). In addition to intra-group differences, intergroup differences were observed across the four protocols (*p* = 0.001). The post-hoc test showed that difference in leg volume were observed in A and C, B and C, and C and D.

Additionally, the difference in both leg volumes between T0-T2 in the MCS-wearing protocols B and D showed a greater decrease compared to leg volumes in not MCS-wearing protocols A and C.

### Leg circumference as a secondary outcome

**Fig. 4:**
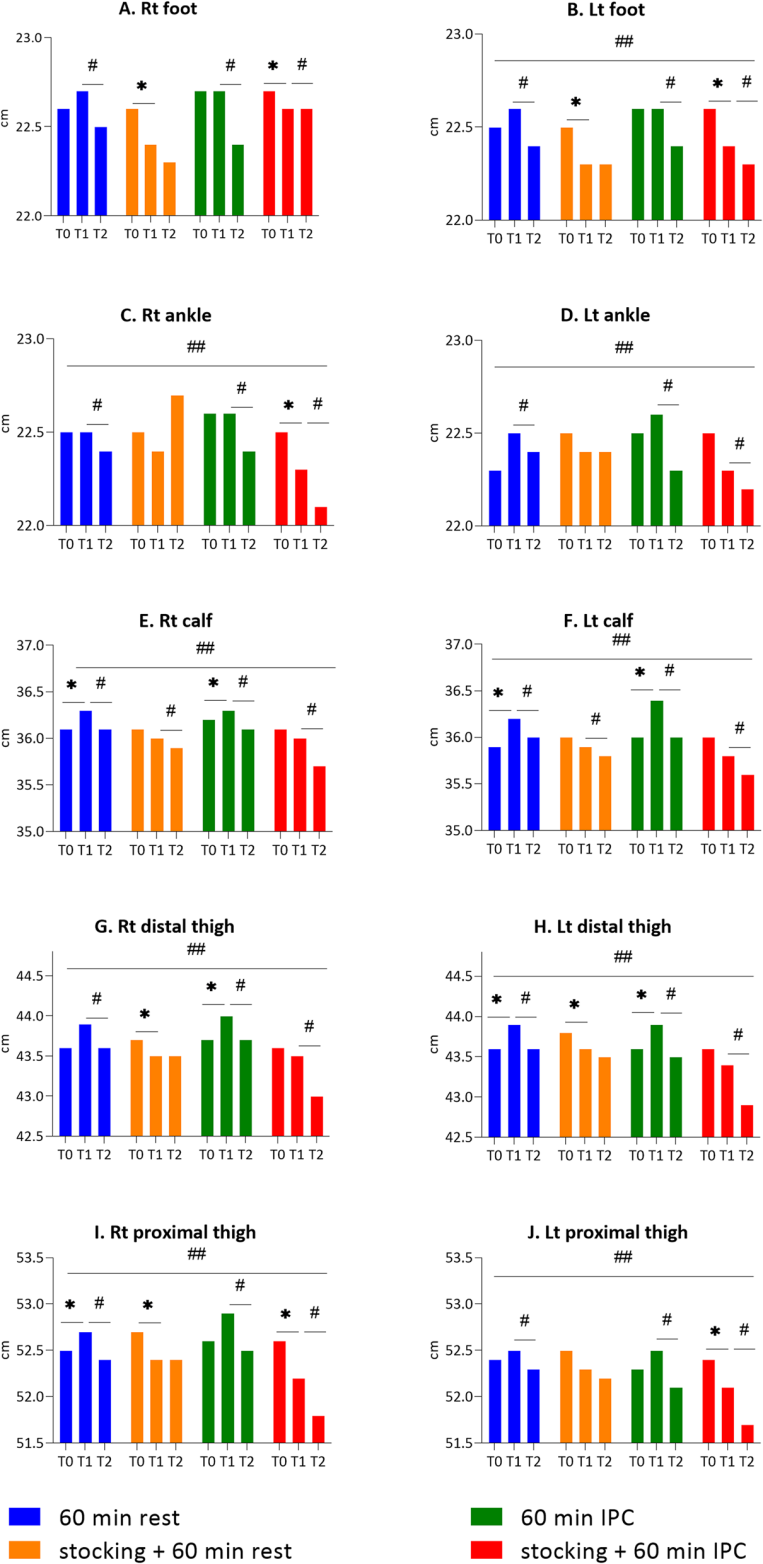
The effect of the different intervention protocols on leg circumference (cm). A. Right foot, B. Left foot, C. Right ankle, D. Left ankle, E. Right calf, F. Left calf, G. Right distal thigh, H. Left distal thigh, I. Right proximal thigh, J. Left proximal thigh, T0: before work, T1: after prolonged standing work, T2: 60 min after intervention *: *p* < 0.05 between T0 and T1, #: *p* < 0.05 between T1 and T2, ##: *p* < 0.05 in the four intervention protocols

Leg circumference measurements at the foot and ankle, 10 cm below the knee, 10 cm above the knee, and 20 cm above the knee in MCS-wearing protocol B and D were reduced in the afternoon after workday (T1) compared to those measured before work (T0), but the circumference measurements in protocols A and C, who did not use MCS, were similar or slightly increased after work (T0-T1) (Fig. 4). The circumference at all measured points after intervention (T1-T2) was significantly decreased in protocol A, C, and D, although those in protocol B did not significantly decrease, with the exception of both calves. When comparing the interventions, there were significant differences across interventions for the circumferences in most of the measured areas except the right foot. In the post-hoc test, there were differences in leg circumference in protocols A and B, B and C, and B and D, at the right ankle, calf, distal thigh, and proximal thigh, and at the left foot, distal thigh, and proximal thigh. In protocols A and C, B and C, and B and D, there were differences in circumference at the left calf, while only in protocols B and C were there any difference at the left ankle (Tables 4 and 5).

**Table 4:** Post hoc results of comparison between the different treatment protocols (A-B, A-C, A-D, B-C, B-D, and C-D) on right leg circumference as a secondary outcome

Protocol	Foot	*p* value (95% CI)	Ankle	*p* value (95% CI)	Calf	*p* value (95% CI)	Distal thigh	*p* value (95% CI)	Proximal thigh	*p* value (95% CI)
A-B	0.12(0.41)	0.083 (-0.01~0.26)	0.40(1.67)	0.022 (-0.14 ~ 0.94)	0.19(0.37)	0.002 (0.08 ~ 0.31)	0.26(0.61)	0.013 (0.06 ~ 0.46)	0.30(0.50)	0.001 (0.14 ~ 0.46)
A-C	-0.05(0.41)	0.289 (-0.18~0.08)	-0.10(0.51)	0.242 (-0.26 ~ 0.07)	0.01(0.40)	0.915 (-0.12 ~ 0.14)	-0.04(0.57)	0.488 (-0.23 ~ 0.14)	-0.04(0.59)	0.516 (-0.23 ~ 0.15)
A-D	0.26(1.66)	0.651 (-0.28~0.80)	-0.05(0.52)	0.300 (-0.22 ~ 0.11)	-0.03(0.33)	0.618 (-0.13 ~ 0.08)	-0.15(0.76)	0.123 (-0.40 ~ 0.10)	-0.04(0.94)	0.944 (-0.34 ~ 0.27)
B-C	-0.17(0.31)	0.001 (-0.28~-0.07)	-0.50(1.83)	0.010 (-1.09 ~ 0.09)	-0.19(0.35)	0.001 (-0.30 ~ -0.07)	-0.31(0.50)	0.001 (-0.47 ~ -0.14)	-0.34(0.48)	< 0.001 (-0.49 ~ -0.18)
B-D	0.13(1.70)	0.175 (-0.42~0.69)	-0.46(1.69)	0.005 (-1.00 ~ 0.09)	-0.22(0.40)	0.002 (-0.35 ~ -0.09)	-0.41(0.62)	< 0.001 (-0.61 ~ -0.21)	-0.33(0.80)	0.029 (-0.59 ~ -0.07)
C-D	0.31(1.64)	0.222 (-0.23~0.84)	0.04(0.51)	0.572 (-0.12 ~ 0.21)	-0.03(0.48)	0.928 (-0.19 ~ 0.12)	-0.11(0.68)	0.401 (-0.33 ~ 0.12)	0.01(0.80)	0.598 (-0.26 ~ 0.27)

Data are presented as mean difference (standard) of leg circumference during T1-T2 between the different treatment groups (cm)

Protocol A, no medical compression stocking (MCS) + natural rest; Protocol B, MCS + natural rest; Protocol C, no MCS + intermittent pneumatic compression (IPC); Protocol D, MCS + IPC

Wilcoxon signed rank test on difference (*p* < 0.008)

**Table 5:** Post hoc results of comparison between the different treatment protocols (A-B, A-C, A-D, B-C, B-D, and C-D) on left leg circumference as a secondary outcome

Protocol	Foot	*p* value (95% CI)	Ankle	*p* value (95% CI)	Calf	*p* value (95% CI)	Distal thigh	*p* value (95% CI)	Proximal thigh	*p* value (95% CI)
A-B	0.23(0.36)	0.001 (0.11 ~ 0.34)	0.11(4.24)	0.089 (-0.03 ~ 0.25)	0.04(0.37)	0.242 (-0.08 ~ 0.16)	0.23(0.42)	0.002 (0.10 ~ 0.37)	0.21(0.41)	0.001 (0.08 ~ 0.34)
A-C	-0.04(0.42)	0.598 (-0.17 ~ 0.10)	-0.12(0.44)	0.043 (-0.26 ~ 0.03)	-0.23(0.41)	0.002 (-0.36 ~ -0.10)	-0.12(0.58)	0.201 (-0.30 ~ 0.07)	-0.19(0.63)	0.082 (-0.40 ~ 0.01)
A-D	0.08(0.39)	0.238 (-0.05 ~ 0.21)	-0.00(0.47)	0.979 (-0.15 ~ 0.15)	-0.09(0.44)	0.276 (-0.24 ~ 0.05)	-0.15(0.68)	0.241 (-0.37 ~ 0.07)	-0.17(0.77)	0.137 (-0.42 ~ 0.08)
B-C	-0.26(0.36)	< 0.001 (-0.38 ~ -0.15)	-0.23(0.38)	0.001 (-0.35 ~ -0.10)	-0.27(0.45)	0.001 (-0.42 ~ -0.12)	-0.35(0.66)	0.001 (-0.57 ~ -0.14)	-0.40(0.49)	< 0.001 (-0.56 ~ -0.24)
B-D	-0.15(0.29)	0.006 (-0.24 ~ -0.05)	-0.11(0.52)	0.167 (-0.28 ~ 0.06)	-0.14(0.36)	0.026 (-0.25 ~ -0.02)	-0.38(0.72)	0.002 (-0.61 ~ -0.15)	-0.38(0.64)	0.001 (-0.59 ~ -0.17)
C-D	0.12(0.39)	0.074 (-0.01 ~ 0.24)	0.12(0.40)	0.065 (-0.01 ~ 0.24)	0.13(0.53)	0.043 (-0.04 ~ 0.30)	-0.03(0.75)	0.749 (-0.27 ~ 0.21)	0.02(0.74)	0.833 (-0.22 ~ 0.26)

Data are presented as mean difference (standard) of leg circumference during T1-T2 between the different treatment groups (cm)

Protocol A, no medical compression stocking (MCS) + natural rest; Protocol B, MCS + natural rest; Protocol C, no MCS + intermittent pneumatic compression (IPC); Protocol D, MCS + IPC

Wilcoxon signed rank test on difference (*p* < 0.008)

### Leg bioelectrical impedance analysis as a secondary outcome

**Fig. 5:**
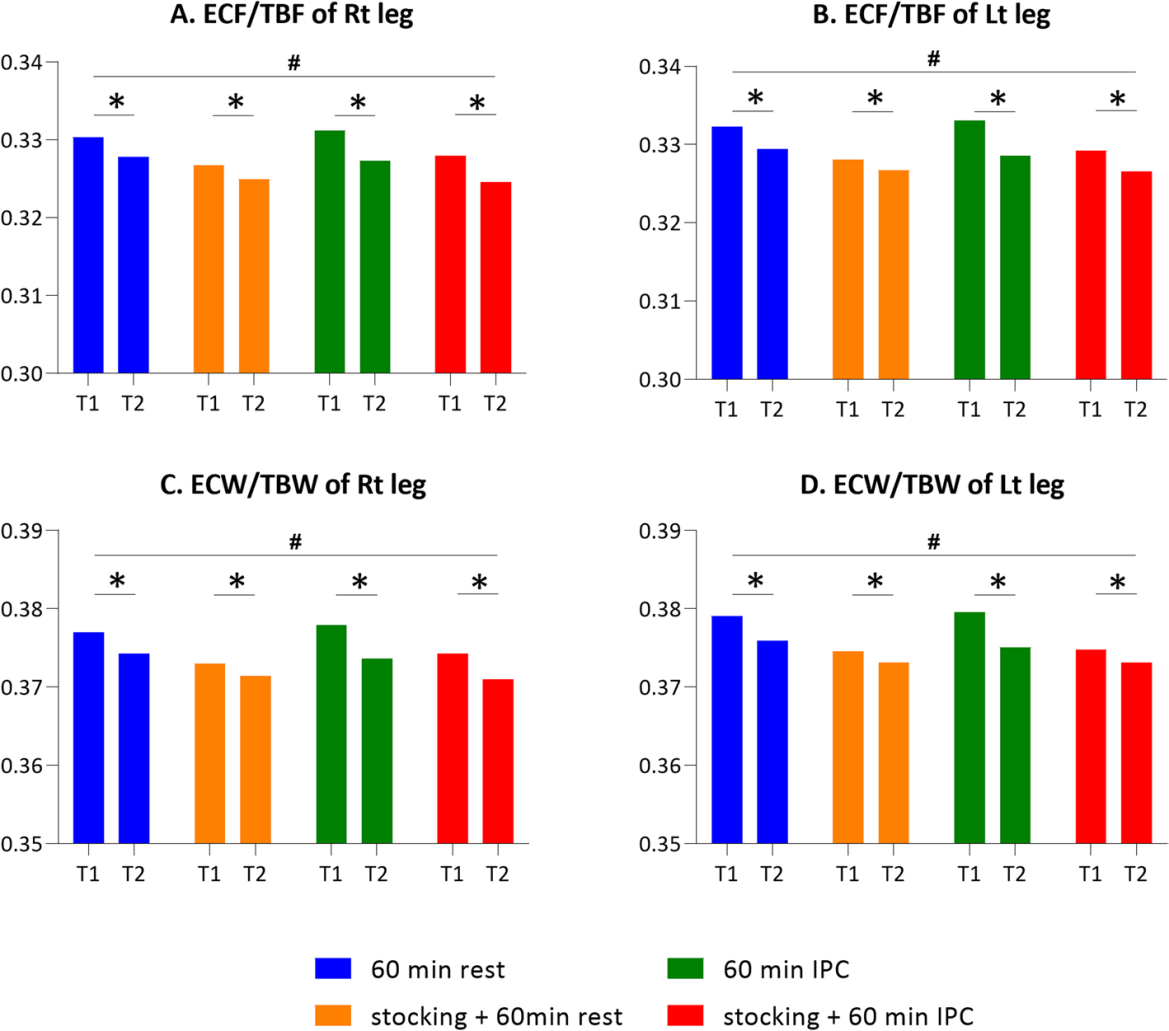
The effect of the different intervention protocols on leg extracellular fluid (ECF)/total body fluid (TBF) and extracellular water (ECW)/total body water (TBW) measured by Inbody system. A. ECF/TBF of Right leg, B. ECF/TBF of Left leg, C. ECW/TBW of Right leg, D. ECW/TBW of Left leg, T1: after prolonged standing work, T2: 60 min after intervention, *: *p* < 0.05 between T1 and T2, #: *p* < 0.05 in the four intervention protocols

Leg ECF/TBF measured by bioelectrical impedance analysis significantly decreased in all interventions bilaterally after the treatment session (T2) compared to those after the workday (T1) (Fig. 5A, B). The ECF/TBF in protocol C treated with IPC showed the largest reduction, and the intergroup difference of ECF/TBF among the four interventions was statistically significant. In the post-hoc test, there were differences observed in all protocols for ECF/TBF of both legs, with the exception of protocols A and D (Table 6).

**Table 6:** Post hoc results of comparison between the different treatment protocols (A-B, A-C, A-D, B-C, B-D, and C-D) on leg extracellular fluid (ECF)/total body fluid (TBF) as a secondary outcome

	Protocol	△ between the groups	*p* value	95% CI
Right	A-B	0.0008(0.0026)	0.008	-0.000 ~ 0.0016
	A-C	-0.0014(0.0021)	< 0.001	-0.0020 ~ -0.0007
	A-D	-0.0007(0.0040)	0.560	-0.0020 ~ 0.0006
	B-C	-0.0022(0.0026)	< 0.001	-0.0030 ~ -0.0013
	B-D	-0.0015(0.0041)	0.005	-0.0028 ~ -0.0002
	C-D	0.0007(0.0043)	0.005	-0.0007 ~ 0.0021
Left	A-B	0.0017(0.0029)	0.001	0.0007 ~ 0.0026
	A-C	-0.0015(0.0025)	< 0.001	-0.0023 ~ -0.0007
	A-D	0.0003(0.0026)	0.665	-0.0005 ~ 0.0012
	B-C	-0.0032(0.0028)	< 0.001	-0.0041 ~ -0.0023
	B-D	-0.0013(0.0030)	0.003	-0.0023 ~ -0.0004
	C-D	0.0018(0.0035)	0.001	0.0007 ~ 0.0030

Data are presented as mean difference (standard deviation)

Protocol A, no medical compression stocking (MCS) + natural rest; Protocol B, MCS + natural rest; Protocol C, no MCS + intermittent pneumatic compression (IPC); Protocol D, MCS + IPC; △, the difference of leg ECF/TBF during T1-T2 between the different treatment protocols

Wilcoxon signed rank test on difference (*p* < 0.008)

Similar to the ECF/TBF, the ECW/TBW significantly decreased in all protocols bilaterally after the intervention (T1-T2), particularly in protocol C (Fig. 5C, D). There were statistically significant differences across the interventions for ECW/TBW. Post-hoc tests showed that the differences between all paired protocols, except A and D, significantly differed in both legs (Table 7).

**Table 7:** Post hoc results of comparison between the different treatment protocols (A-B, A-C, A-D, B-C, B-D, and C-D) on leg extracellular water (ECW)/total body water (TBW) as a secondary outcome

	Protocol	△ between the groups	*p* value	95% CI
Right	A-B	0.0011(0.0029)	0.004	0.0002 ~ 0.0020
	A-C	-0.0015(0.0023)	< 0.001	-0.0022 ~ -0.0007
	A-D	-0.0005(0.0042)	0.921	-0.0019 ~ 0.0009
	B-C	-0.0026(0.0027)	< 0.001	-0.0034 ~ -0.0017
	B-D	-0.0016(0.0044)	0.005	-0.0030 ~ -0.0002
	C-D	0.0009(0.0044)	0.001	-0.0005 ~ 0.0024
Left	A-B	0.0017(0.0030)	0.001	0.0008 ~ 0.0027
	A-C	-0.0015(0.0027)	0.001	-0.0023 ~ -0.0006
	A-D	0.0015(0.0068)	0.266	-0.0007 ~ 0.0037
	B-C	-0.0032(0.0029)	< 0.001	-0.0042 ~ -0.0023
	B-D	-0.0002(0.0077)	0.010	-0.0027 ~ 0.0023
	C-D	0.0030(0.0075)	0.001	0.0005 ~ 0.0054

Data are presented as mean difference (standard deviation)

Protocol A, no medical compression stocking (MCS) + natural rest; Protocol B, MCS + natural rest; Protocol C, no MCS + intermittent pneumatic compression (IPC); Protocol D, MCS + IPC; △, the difference of leg ECW/TBW during T1-T2 between the different treatment protocols

Wilcoxon signed rank test on difference (*p* < 0.008)

### Safety events

Although we created a comprehensive system for monitoring and recording adverse events potentially occurring during this study, no serious adverse events were noted in the study population.

## Discussion

This study confirmed that applying IPC in prolonged standing workers significantly reduced pain and swelling compared to wearing MCS. In addition, when working in a standing position all day long, there was no significant difference in pain regardless of whether or not MCS were worn. During prolonged standing work wearing MCS for 8 h, the leg volume was reduced immediately after working (T1), but the pain was induced similarly to when it is not worn. Leg circumference was measured at the foot, ankle, calf, distal thigh, and proximal thigh, and except for protocol B wearing MCS only, there was a statistically significant decrease after therapy, especially in the proximal area. Since there was no decrease in circumference nor significant difference immediately after working in protocol B, the effect of the therapy was relatively small compared to other protocols, and there was no significant decrease except for the measurements at both calves. The reason that there was a significant difference between protocol B and other protocols in the post-hoc test was that there was no increase in leg circumference at T1 in protocol B; hence, the significant difference may appear from other protocols whose leg circumference increased after working in the afternoon. ECF/TBF and ECW/TBW, which are indicators of leg edema, showed statistically significant decreases after the therapy in all protocols. In particular, protocol C, which applied IPC alone, showed the most significant decrease. Therefore, the findings of this study confirmed that IPC can effectively treat leg pain and edema in people with occupations requiring prolonged standing. Although several studies have reported on the effectiveness of IPC and MCS in people who were standing for a long time or patients with venous insufficiency, this study contained several unique issues [6, 15–18, 21, 24, 34]. First, this study targeted people who had an 8 h standing occupation rather than a short standing or sedentary occupation. Second, the Duplex ultrasound was performed as a screening test for people without underlying venous disease to determine obstructive venous disease. Third, the effectiveness of each therapy on parameters and symptoms was confirmed by comparing single and combined protocols of IPC and MCS. Finally, ECF/TBF and ECW/TBW were also confirmed using bioelectrical impedance analysis, as well as leg swelling parameters such as volume and circumference, which have been commonly used in previous studies.

A previous study of people without any specific vascular diseases or symptoms with prolonged standing occupations reported that the ankle hydrostatic venous pressure increased to 90 mmHg and leg volume deteriorated to 50 mL when standing for a long time [30–33]. Similar to previous studies, this study confirmed that prolonged standing work causes leg pain and swelling [2–5, 24, 30–34]. In protocol A and C where stockings were not worn, the mean leg volume at T1 compared to that at T0 increased by 65.4–81.1 mL, and the circumference increased by 0–0.1 cm in the distal area below the calf and clearly increased by 0.1–0.4 cm in the proximal area. Prolonged standing work causes leg swelling and chronically increases hydrostatic venous pressure, leading to venous retention and venous reflux. In addition, venous reflux ultimately causes venous insufficiency, leading to secondary complications such as venous ulcers. In a previous study, venous reflux was found in more than 80% of ultrasound tests performed on healthcare workers [1]. In the duplex ultrasound screening test in this study, venous reflux was found in 89.7% of healthy subjects, showing results similar to the previous study [1]. Thus, if an individual’s occupation involves prolonged standing and he/she has venous symptoms in the lower limb, the risk of venous insufficiency is high and needs to be assessed.

One of the most common therapies for venous insufficiency is compression therapy, which provides graded external compression of the legs and prevents lower limb hypertension [35, 36]. MCS is the most commonly used compression garment, and although different pressures are recommended depending on the severity, approximately 20–50 mmHg of pressure should be applied. Wearing MCS with approximately 30–40 mmHg of pressure is known to be helpful for pain, swelling, skin pigmentation, activity, and well-being [37]. It has been reported that wearing MCS in patients with CVI significantly reduces leg pain, swelling, skin discoloration, activity tolerance, depression, and sleep problems [29, 37–40]. Complete ulcer healing has been reported after wearing MCS for an average of 5.3 months in severe CVI patients with a venous ulcer [41], and a hemodynamic benefit to reduce reflux in residual volume fractional vein segments has been reported [42, 43]. However, previous studies were insufficient to compare the effects of MCS and other therapies for pain and swelling, and the effects of resting. When comparing protocol A (resting) and protocol B (resting + MCS) in this study, the pain scores measured before and immediately after working increased in both protocols, and both pain scores decreased after natural resting. However, there was no statistical difference between the two protocols in the post-hoc test. The volume was measured before and immediately after working, and swelling was less induced in protocol B than in protocol A. The volume measured after the intervention (T2) did not significantly differ between protocols A and B at the post-hoc test. The other leg swelling indicators (circumference, ECF/TBF, and ECW/TBW) also showed similar trends. Protocol D, using both MCS and IPC, experienced a statistically significant effect on pain and swelling, but there was no difference with protocol C in the post-hoc test. As a result, this study showed that wearing MCS while working reduced swelling immediately after working, but there was no effect on pain reduction and swelling compared to IPC only as intervention. Additionally, the effect of the natural resting therapy tended to increase when wearing MCS, but it was not statistically significant, and wearing MCS did not affect IPC therapy.

IPC, which is a form of compression therapy, uses a machine that can be applied with various pressures and modes to the lower limb, and its effects and principles have been reported in several studies. According to previous studies, when IPC is applied to the feet, the peak velocity of the common femoral vein is increased; when IPC of 120–180 mmHg is applied to the lower limb below the knee, the venous flow velocity and pulsatility index are increased. It also causes the largest increase in venous outflow in the foot and calf [44]. The risk for blood vessel obstruction with IPC use has been reported, but the application of IPC to healthy subjects increases the oxygenation of muscle cells in the lower limb [45]. Like MCS, there are still no international consensus pressure values or modes in IPC. In the evaluation of the stability and efficacy of IPC therapy in 19 healthy subjects, which was conducted before this study, a statistically significant reduction in leg pain and edema was observed when performing 30 min of IPC at 90–120 mmHg and 30 min of natural resting, and there were no adverse effects. In addition, both the circular and sequential modes were effective without a statistical difference. Based on this, IPC intervention in this study was performed for a total of 60 min in circular and sequential modes for 30 min each at 90–120 mmHg pressure; protocol C showed the greatest reduction in leg pain and edema. Furthermore, when observing the synergistic effect of wearing MCS during work and IPC intervention, this study showed an apparent decrease in edema immediately after working (T0-1), but there was no improvement in pain immediately after working and after the therapy (T0-1, 1–2), nor in edema after the therapy (T1-2). The effect of the compression garment was increased by maintaining pressure along with exercise or massage treatment, but the previously reported effects of MCS were not shown, since rest or exercise was avoided as much as possible and MCS was worn only in a situation where pain and swelling were induced. Alternatively, if the pressure of MCS is modified (high or low), it may be effective in reducing pain or swelling; thus, further research is needed.

Although the statistical power was 0.84, this study had a relatively small number of participants, compared to the previous studies. However, significant differences were observed related to leg pain and edema. Additionally, all participants did not do the same standing work, such as giving lectures, a cashier’s job, or caring for hospitalized patients. In the case of the occupational group that includes both static standing and walking, such as nurses, it might affect pain and swelling compared to the group that mainly performs static standing, for example, cashiers. The distance and methods of the visits to the center differed for the participants. These variables might indirectly affect the therapeutic effect. Furthermore, the sequences of the four intervention protocols were not randomized and selected, which might have affected the reporting bias. Moreover, the types of socks worn, while not wearing MCS, were not fully controlled, which could also be another study limitation. The other limitation is that the pressure of MCS was set to 23–32 mmHg, which has reportedly been the recommended pressure. As mentioned above, the optimal setting of MCS is still controversial, and MCS intervention with a high pressure of over 33 mmHg might significantly reduce leg pain and edema or show a synergistic effect with IPC therapy.

## Conclusions

This study suggested more effective treatment combinations of resting, MCS, and IPC for each of the symptoms of leg pain and edema after working in a prolonged standing position. Resting and not wearing MCS, resting and wearing MCS, IPC and not wearing MCS, and IPC and wearing MCS significantly improved leg pain, especially IPC and not wearing MCS. In addition, wearing MCS protocol prevented leg edema during the workday. Additionally, the presence of leg edema after working in a prolonged standing position in not wearing MCS protocol decreased after IPC interventions.

## Data Availability

All data generated or analyzed during this study are included in this published article and its supplementary information files.

## Abbreviations

MCS: Medical compression stockings
IPC: Intermittent pneumatic compression
VAS: Visual analogue scale
ECF: Extracellular fluid
TBF: Total body fluid
ECW: Extracellular water
TBW: Total body water
CVI: Chronic venous insufficiency
CEAP: Clinical, Etiology, Anatomy, and Pathophysiology
RM-ANOVA: Repeated measures analysis of variance
